# Self Esteem among Tunisian Adolescents: Modulating factors

**DOI:** 10.1192/j.eurpsy.2023.1214

**Published:** 2023-07-19

**Authors:** H. Rezgui, S. Bourgou, R. Gadhoum, H. ben youssef, M. hamza, S. Hadj amor, R. Fakhfekh, A. Belhadj

**Affiliations:** 1 Child and Adolescents psychiatry, Mongi Slim Hospital, tunis, Tunisia; 2 ministry of health, tunis; 3 Abderrahmen Mami Hospital, tunis, Tunisia

## Abstract

**Introduction:**

Self-esteem is a valuable personal asset and it has shown to be related to well-being across cultures and nations.

**Objectives:**

Explore the level of self-esteem among Tunisian adolescents and the different factors that modulate it.

**Methods:**

We conducted a cross sectional study among adolescents who attend Tunisian high school from 11 October 2021 to 11 November 2021. Adolescents filled the Tunisian version of Body Esteem Scale of Adolescents and Adults, the Arabic version of the Rosenberg Self Esteem Scale and a questionnaire containing socio- demographic and clinical variables.

**Results:**

The population was made of 60.3% of girls and 39.7% of boys. Their age varied from 12 to 19 years’ old. The adolescents were attending the high school from seventh to third year of secondary school. Their body mass index (BMI) was normal in 58.2%, <18.5 in 31.5% and ≥ 25 in 10.3%. the score of the Rosenberg Self Esteem Scale ranged from 13 to 40 with an average of 31.02.

Female adolescents had a low self-esteem (Rosenberg Self Esteem Score <31) in 59.51%, while only 39.25% of male adolescents had a low self-esteem.

We did not find significant correlation between self-esteem and age. We found positive correlation between self-esteem and body esteem (r= 0,422; p <0.01) and a negative correlation between self-esteem and body mass index (r= -0,131; p <0,05). We found that adolescents with high self-esteem were more satisfied with their weight, their appearance and that they perceived a good external evaluation. We found also that when body mass index increased, self-esteem decreased.

**Conclusions:**

It’s widely important to study self-esteem among adolescents because it’s modulated by several factors and a low self-esteem may have a negative impact on the mental health.

**Disclosure of Interest:**

None Declared

